# Editorial Department

**Published:** 1855-10

**Authors:** 


We extract from the Boston Medical Journal, the following touching tribute to the momory of Prof. Elisha Bartlett, whose death we announced in our last. It is from the eloquent pen of Dr. O. W. Holmes:

Everywhere through his writing prevails that easy flow of language, that felicity of expression, that florid warmth when occasion offers, which commonly marks the prose of those who are born poets. Yet few suspected him of giving utterance in rhythmical shape to his thought or feelings. It was only when his failing limbs could bear him no longer, as conscious existence slowly retreated from their palsied nerves, that he revealed himself freely in this truest and tenderest form of expression. We knew that he was dying by slow degrees, and we heard from him from time to time, or saw him, always serene and always hopeful while hope could have a place in his earthly future. His work was done, done nobly and gracefully, the work of an honest citizen, of a revered teacher, of a wise thinker. When to the friends he had loved, there came as a farewell gift not a last effort of the learning and wisdom they had been taught to expect from him, but a little book with a few songs in it, songs with his whole warm heart in them, they knew that his hour was come, and their tears fell fast as they read the loving thoughts that he had clothed in words of natural beauty and melody. The cluster of evening primroses had opened, and the night was close at hand.

No brief tribute like this can do more than show the feelings which its subject inspired in those who knew him. He has left this earthly scene of his labors too early for friendship and for science, not for himself, ripe in every virtue, and ready for wider spheres of knowledge; one of the "pure in heart," who look on the unveiled face of truth during their earthly pilgrimage, and who have the promise that they shall "see God" himself when they have reached its close. O. W. H.

